# The additive effects of bioactive glasses and photobiomodulation on enhancing bone regeneration

**DOI:** 10.1093/rb/rbad024

**Published:** 2023-03-24

**Authors:** Lidong Huang, Weiyu Gong, Guibin Huang, Jingyi Li, Jilin Wu, Yanmei Dong

**Affiliations:** Department of Cariology and Endodontology, Peking University School and Hospital of Stomatology & National Center for Stomatology & National Clinical Research Center for Oral Diseases & National Engineering Research Center of Oral, Biomaterials and Digital Medical Devices, Beijing 100081, China; Department of Cariology and Endodontology, Peking University School and Hospital of Stomatology & National Center for Stomatology & National Clinical Research Center for Oral Diseases & National Engineering Research Center of Oral, Biomaterials and Digital Medical Devices, Beijing 100081, China; Department of Cariology and Endodontology, Peking University School and Hospital of Stomatology & National Center for Stomatology & National Clinical Research Center for Oral Diseases & National Engineering Research Center of Oral, Biomaterials and Digital Medical Devices, Beijing 100081, China; Department of Cariology and Endodontology, Peking University School and Hospital of Stomatology & National Center for Stomatology & National Clinical Research Center for Oral Diseases & National Engineering Research Center of Oral, Biomaterials and Digital Medical Devices, Beijing 100081, China; Department of Cariology and Endodontology, Peking University School and Hospital of Stomatology & National Center for Stomatology & National Clinical Research Center for Oral Diseases & National Engineering Research Center of Oral, Biomaterials and Digital Medical Devices, Beijing 100081, China; Department of Cariology and Endodontology, Peking University School and Hospital of Stomatology & National Center for Stomatology & National Clinical Research Center for Oral Diseases & National Engineering Research Center of Oral, Biomaterials and Digital Medical Devices, Beijing 100081, China

**Keywords:** bioactive glasses, photobiomodulation, osteogenesis, angiogenesis, tissue engineering

## Abstract

Bioactive glasses (BG) have been generally used in bone defects repair for its good osteoinductivity and osteoconductivity. However, the early angiogenesis of BG in the repair of large-sized bone defects may not be sufficient enough to support new bone formation, resulting in the failure of bone repair. Photobiomodulation (PBM) therapy, which is superior on promoting early angiogenesis, may contribute to the angiogenesis of BG and further enhance the repair of bone defects. Therefore, we applied BG and PBM in combination and preliminarily investigated their additive effects on bone regeneration both *in vitro* and *in vivo*. The *in vitro* results revealed that BG combined with PBM remarkably enhanced human bone marrow mesenchymal stem cells proliferation, osteogenic-related genes expression and mineralization, which was better than applying BG or PBM respectively. For *in vivo* studies, the histological staining results showed that BG induced new bone formation in the interior of defects and promoted new bone reconstruction at 6 weeks post-operation. The micro-computed tomography results further confirmed that BG combined with PBM accelerated bone formation and maturation, improved the speed and quality of bone regeneration, and promoted bone repair. In conclusion, with the optimum BG and PBM parameters, BG combined with PBM generated additive effects on promoting bone regeneration.

## Graphical Abstract

**Figure:**
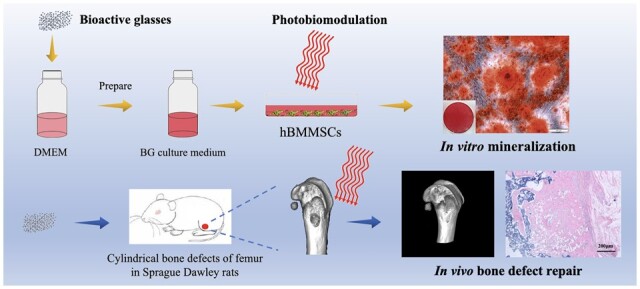

## Introduction

Biomaterials, as one of the three elements of tissue engineering, provide good inducing environment for cells’ adhesion, proliferation and differentiation, which is a critical factor for the success of tissue regeneration [1]. Bioactive glass (BG) is a kind of bone grafting material with superior bioactivity and osteoinductivity [2]. After contacting with the tissue fluid, BG particles release silicon (Si), calcium (Ca) and phosphorus (P) ions. The ions can activate several signal pathways, such as the mitogen-activated protein kinase (MAPK) pathway [3–6], up-regulate the expression of osteogenic-related genes [4, 7, 8], and promote the proliferation and differentiation of osteoblasts [9]. Compared with hydroxyapatite, tricalcium phosphate and some other materials, BG can stimulate the differentiation of surrounding mesenchymal stem cells into osteoblasts, not only supporting the growth of new bone at the interface between the bone defect tissue and the material, but also inducing bone formation inside the material and more distally [10]. BG has been widely applied and achieved good clinical effects [2].

However, in the repair of critical-sized bone defects with implantation of biomaterials, bone neoformation in the central area may be difficult because of the insufficient synchronous vascular network restoration in the early stage, which mainly due to the size of defects, the morphology of biomaterials as well as the rate and concentration of the ion release. A previous study [11] showed that nanosized BG did not reveal obvious promoting effect on endothelial cell migration in the first 5 hours, which may be correlated with the inadequate synthesis of some pro-angiogenic growth factors and their receptor proteins. Therefore, it might be suggested that the early angiogenesis of BG was insufficient [11]. The delayed endothelial cells migration and inadequate early angiogenesis often lead to the insufficient supply of nutrients and detrimental changes in microenvironment, further resulting in tissue ischemia and necrosis, and consequently hindering the bone regeneration [12]. Therefore, solving the problem of inadequate early angiogenesis in large-sized bone defects repaired by biomaterials is an important topic in tissue engineering.

Photobiomodulation (PBM) utilizes low-level energy to elicit photophysical and photochemical reactions, as well as generate biostimulatory effects [13]. In the early stage of tissue regeneration, PBM can promote the synthesis of vascular endothelial nitric oxide synthase, increase the production of pro-angiogenic growth factors, enhance the migration and proliferation of endothelial cells, and facilitate angiogenesis [14–17]. PBM also plays a positive role in promoting bone regeneration and accelerating wound healing [18, 19]. Therefore, PBM therapy has been considered as a good method to promote the early angiogenesis, which may exert beneficial effects on the repair of critical-sized bone defects. However, PBM also has some limitations. Firstly, the penetration depth of lights is limited. The biostimulatory effect decreases as the depth of tissue increases, which makes PBM inappropriate for the deep bone defects [20]. Secondly, the PBM’s biostimulatory effect is dose-dependent. Only the appropriate doses induce cell activity. Neither low nor high doses could produce the promoting effects [21]. Also, PBM cannot provide physical support and continuous biological stimulation for tissues regeneration. Therefore, biomaterials are still indispensable in promoting bone defects repair and playing a long-term role.

Based on the respective characteristics of BG and PBM, we hypothesized that with the combined application of BG and PBM, there would be additive effects on the enhancement of bone regeneration. Our previous study indicated that the combination of BG and PBM could additively promote the early angiogenesis [22]. In this research, we explored the effects of BG in combination with PBM on human bone marrow mesenchymal stem cells’ (hBMMSCs) proliferation, osteogenic-related genes expression and mineralization *in vitro*, as well as their additive effects on bone defects repair *in vivo*, aiming at investigating the osteogenic effect of their combined application.

## Materials and methods

### Preparation of BG particles and BG culture medium

The BG utilized in the research was named as PSC, which was synthesized by the sol–gel technique with phytic acid as phosphorus precursor [23, 24]. The composition of PSC was 22.7% P_2_O_5_, 48.2% SiO_2_ and 29.1% CaO (wt%). The granules were irregularly shaped, with an average diameter of 21 ± 12 μm and a specific surface area of 53.5 m^2^/g. BG particles were sterilized and added into Dulbecco’s modified Eagle medium (DMEM, Gibco, Waltham, MA, USA). Suspensions were shaken for 24 h at 37°C, and then filtered and prepared into ionic extracts. Then, add 10% (in volume percent) fetal bovine serum, 1% l-glutamine and 1% penicillin–streptomycin to the ionic extracts for the preparation of BG culture medium.

### Application of PBM therapy

PBM therapy was applied using an 808 nm-wavelength near-infrared (NIR) diode laser (Beijing Laserwave Optoelectronics Tech, Co., Ltd). The specific parameters of PBM therapy applied throughout the study were shown in Table 1. In *in vitro* study, adjust the distance of the laser tip and the culture dish to determine the irradiance at 50 mW/cm^2^. Then, the fluence that cells received was determined by the irradiation time. The untreated wells were covered with aluminum foil.

**Table 1.** The parameters of PBM therapy

Parameters	MTT assay	Real-time RT-PCR and alizarin red staining	*In vivo*
Mode	CW	CW	CW
Irradiance (mW/cm^2^)	50	50	200
Fluence (J/cm^2^)	0.5, 1, 3, 5	3^a^	120
Time of irradiation (s)	10, 20, 60, 100	60	600
Spot size (cm)	4^b^	4^b^	1.3
Distance of tip and tissues (cm)	9.8	9.8	2

CW, continuous-wave.

a The fluence of PBM in real-time RT-PCR and alizarin red staining was the optimum fluence identified by the MTT assay.

b The actual spot size in *in vitro* study should be determined by the size of the window on aluminum foil.

### Cell culture

Primary hBMMSCs (PromoCell, Heidelberg, Germany) were cultivated in DMEM added with 10% (in volume percent) fetal bovine serum, 1% penicillin–streptomycin and 1% l-glutamine at 37°C with 5% CO_2_. Change the medium every 2 days. The hBMMSCs of passages 4–6 were used in the following study.

### Proliferation assay of the hBMMSCs

#### Proliferation of hBMMSCs cultured in different concentrations of BG culture medium by the 3-(4,5-dimethylthiazol-2-yl) 2,5-diphenyltetrazolium bromide (MTT) assay

hBMMSCs were seeded at 3 × 10^3^ cells/well into 96-well plates with five duplicate samples for each group. After 24-h incubation, replace the medium with BG culture medium of different concentrations (0.01, 0.1, 1 and 2 mg/ml). As for the control group, replace the medium with DMEM without BG extracts. The timepoint was recorded as Day 0. The culture medium was changed every 2 days. hBMMSCs proliferation was detected on Days 1, 3, 5, 7 and 10 by the MTT (Sigma-Aldrich, St. Louis, MO, USA) assay. The optical density (OD) value was measured at 490 nm by a microplate reader. After statistical analysis revealed the group that displayed the most enhancing cells proliferation, the optimum BG concentration for hBMMSCs growth was selected for subsequent experiments.

#### Proliferation of hBMMSCs treated with different fluences of PBM by the MTT assay

To confirm the optimum fluence of the PBM, hBMMSCs were seeded at 3 × 10^3^ cells/well in 96-well plates with five duplicate samples for each group. After 24-h incubation, cells were irradiated by PBM with different fluences (0.5, 1, 3 and 5 J/cm^2^) (Table 1). The plate was covered with aluminum foil to make sure that the light was delivered to the single well each time. The proliferation of hBMMSCs without PBM treatment was assayed as the control group. The timepoint was recorded as Day 0. PBM therapy was conducted on Days 0, 1 and 2. Change the culture medium every 2 days. The hBMMSCs proliferation was detected on Days 1, 3, 5, 7 and 10 by MTT assay. After statistical analysis revealed the group of fluence that displayed the most enhancing effect on cells proliferation, the optimum fluence of PBM for hBMMSCs growth was selected for subsequent experiments.

#### Proliferation of hBMMSCs exposed to BG combined with PBM by the MTT assay

After the optimum concentration of BG and the optimum fluence of PBM were identified by the experiments above, the hBMMSCs were divided into the following groups: BG + PBM (hBMMSCs cultured in BG culture medium and received PBM in the first 3 days); PBM (hBMMSCs cultured in DMEM and received PBM in the first 3 days); BG (hBMMSCs cultured in BG culture medium) and the control group (hBMMSCs cultured in DMEM). hBMMSCs were seeded in 96-well plates and each group had five replicates. After 24-h incubation, the medium of the BG + PBM group and the BG group was replaced with BG culture medium of the optimum concentration. The timepoint was recorded as Day 0. Change the medium every other day. The BG + PBM and PBM groups were treated by PBM under the optimum fluence on Days 0, 1 and 2. The irradiance of PBM therapy was set at 50 mW/cm^2^, and the fluence received was determined by the irradiation time (Table 1). The hBMMSCs proliferation was detected on Days 1, 3, 5, 7 and 10 by the MTT assay.

### Expression of osteogenic-related genes by real-time reverse transcription-polymerase chain reaction

The hBMMSCs were seeded into six-well plates at 1.5 × 10^5^, 1 × 10^5^ and 0.8 × 10^5^ cells/well, respectively. The group of experiments and the treatment of cells were the same as the above experiment. On Days 0, 1 and 2, PBM therapy was carried out in the wells of the BG + PBM group and the PBM group (Table 1). The mRNA expression was detected on Days 2, 4 and 7. Total RNA was isolated using the TRIzol reagent (Invitrogen, Waltham, MA, USA) following the manufacturer’s instructions. Then, cDNA was synthesized by the Prime Script RT Master Mix (Takara, Tokyo, Japan). The target genes were ALP, Col-I, Runx2 and OCN. The gene of glyceraldehyde 3-phosphate dehydrogenase (GAPDH) was used as endogenous reference. The primer’s sequences were shown in Table 2. Polymerase chain reaction (PCR) amplification was performed using FastStart Universal SYBR Green Master reverse transcription kit (Roche, Indianapolis, IN, USA). All reactions started with pre-denaturation at 95°C for 10 min, followed by denaturation at 95°C for 15 s and annealing/extending at 60°C for 1 min for 40 cycles. The 2^−ΔΔCt^ method was used for data analysis.

**Table 2.** The primers sequences for real-time RT-PCR

Primers	Sequences
ALP	Forward: 5′-AGCACTCCCACTTCATCTGGAA-3′
	Reverse: 5′-GAGACCCAATAGGTAGTCCACATTG-3′
Col-I	Forward: 5′-CGAAGACATCCCACCAATCAC-3′
	Reverse: 5′-TGTCGCAGACGCAGAT-3′
Runx2	Forward: 5′-ACCCAGAAGGCACAGACAGAAG-3′
	Reverse: 5′-AGGAATGCGCCCTAAATCACT-3′
OCN	Forward: 5′-AGGGCAGCGAGGTAGTGA-3′
	Reverse: 5′-CCTGAAAGCCGATGTGGT-3′
GAPDH	Forward: 5′-GAAGGTGAAGGTCGGAGTC-3′
	Reverse: 5′-GAGATGGTGATGGGATTTC-3′

### Mineralization of hBMMSCs by alizarin red staining assay

The osteogenic medium (OM) was prepared by adding 10% (in volume percent) fetal bovine serum, 1% penicillin–streptomycin, 1% l-glutamine, 1% vitamin C, 1% β-sodium glycerophosphate and 0.2% dexamethasone into DMEM. The 7.4% sodium bicarbonate was added to adjust the pH value to the range of 7.0–7.4. The group of experiments were as follows: BG + PBM + OM group (hBMMSCs cultured with BG culture medium supplemented with OM and received PBM in the first 3 days); PBM + OM group (hBMMSCs cultured in DMEM supplemented with OM and received PBM in the first 3 days); BG + OM group (hBMMSCs cultured with BG culture medium supplemented with OM); OM group (hBMMSCs cultured in DMEM supplemented with OM) and control group (hBMMSCs cultured in DMEM).

hBMMSCs were seeded in the six-well plate with 5 × 10^4^ cells/well, and then incubated at 37°C and 5% CO_2_. After 24-h incubation, replace the medium according to the above group arrangement. The timepoint was recorded as Day 0. Change the medium every other day. On Days 0, 1 and 2, the BG + PBM + OM group and PBM + OM group were treated with PBM. On Days 14 and 21, alizarin red staining was conducted. Fix the cells with 2 ml 4% paraformaldehyde for 15 min, and use milli-Q water to wash the cells for three times. Then, 40 mM alizarin red solution was used to stain the samples for 20 min, and then use milli-Q water to rinse samples and remove the non-specific staining. Inverted phase contrast microscope (TE2000-U, Nikon, Japan) was used for observation. After staining, 1 ml 100 mM cetylpyridinium chloride solution was added to each well to dissolve the staining. The quantitative analysis was performed by measuring the OD values at 562 nm by the microplate reader.

### Observation of new bone formation *in vivo*

#### Experimental grouping and animal surgeries

Twenty-eight healthy 12-week-old Sprague Dawley rats, weighing around 500 g, were provided by Beijing Vital River Laboratory Animal Technology Co., Ltd. The research scheme was approved by the ethical review of the Biomedical Ethics Committee of Peking University (LA2016309). The observing timepoints were arranged at 6- and 12 weeks post-operation. Twelve rats were randomly chosen as the experimental animals of the 6-week timepoint groups. The total 24 femur samples of the 12 rats were randomly assigned, with 6 samples to each group respectively (BG + PBM group, PBM group, BG group and control group). The rest 16 rats were divided into the 12-week groups, and the total 32 femur samples were randomly assigned, with 8 samples in each group.

Firstly, rats were anesthetized intraperitoneally with 5% chloral hydrate (0.7 ml/100 g). Subsequently, an incision of 2 cm long was made on the lateral side of the knee joint. The distal metaphysis of femur was exposed by separating subcutaneous fascia and muscles. Then, a cylindrical defect with 3 mm in diameter and 3 mm in depth was created at the metaphysis. PBM therapy was performed in the groups of BG + PBM and PBM. Fix the vertical distance between the laser and the surface at 2 cm and make sure the light spot completely covered the bone defect. The irradiation was set at 200 mW/cm^2^ and the irradiation time was 10 min. In total, bone defects actually received 120 J/cm^2^ of fluence each time (Table 1). PBM therapy was conducted three times, which was before BG particles implanted, the first- and second-day post-operation. After the first irradiation, the defects of BG-treated groups were filled with BG particles and the defects in other groups were filled only with blood clots. After that, incisions were closed. On Days 1 and 2 post-operation, PBM was performed with a 24-h interval between treatments.

#### Histological changes observed by hematoxylin–eosin staining and Masson staining

At 6 weeks post-surgery, the animals of 6-week groups were euthanized under excessive anesthesia. Samples were taken and embedded in paraffin. Then, sections were sliced perpendicular to the surface of bone defect and parallel with the femoral long axis, and the thickness of each slice was 5 μm. Slices were serial numbered and equidistantly selected, and then dewaxed in xylene and rehydrated with ethanol.

For hematoxylin–eosin (HE) staining, slices were stained with hematoxylin for 15 min and washed off the floating color by running water. One percent of hydrochloric acid alcohol was utilized to differentiate the slices. Subsequently, slices were washed and stained with eosin for 1 min. Then, slices were successively immersed in graded ethanol and cleared by xylene.

For Masson staining, slices were stained with the mixed solution of hematoxylin A solution and hematoxylin B (in a ratio of 1:1) for 7 min. Then, the floating color was washed with flowing water. Slices were differentiated for 5 s by 1% hydrochloric acid alcohol, rinsed by distilled water for 5 min and stained with ponceau acid fuchsin for 6 min. Then, the slices were rinsed for 1 min with the weak acid working solution, which was made up of weak acid and distilled water at a ratio of 1:2. After the rinse, the slices were stained with molybdophosphoric acid for 5 min and washed in weak acid working solution for 1 min. After stained with aniline blue for 5 min and washed with weak acid working solution for 1 min, slices were immersed in ethanol of gradient concentrations and cleared in xylene.

#### New bone formation measured by micro-computed tomography

At 6 and 12 weeks after the surgery, the animals of 6- and 12-week groups were euthanized under excessive anesthesia and the specimens were fixed in 10% formalin for 48 h. For micro-computed tomography (micro-CT) scanning, the voltage was 80 kV, the current was 500 μA and the exposure time was 1500 ms. The specimen was rotated 360° and scanned every 1°. Then, the original data of each specimen were reconstructed by the software COBRA Exxim.

The 3D images were analyzed by the Inveon Acquisition Workplace software. Adjust the image to place the bone defect horizontally. A cylinder with the diameter of 3 mm and the depth of 3 mm perpendicular to the surface of defect was set as region of interest. Then, the bone volume/tissue volume (BV/TV), bone mineral density (BMD), trabecular number, trabecular thickness and trabecular spacing were statistically analyzed.

### Statistical analysis

All *in vitro* studies were performed at least three independent experiments. SPSS 22.0 was used for statistical analysis. One-factor ANOVA was performed to evaluate the statistical significance of experimental results. The LSD test was used for groups comparison. *P *<* *0.05 indicated statistical significance.

## Results

### BG combined with PBM promoted hBMMSCs proliferation

**Figure 1.**
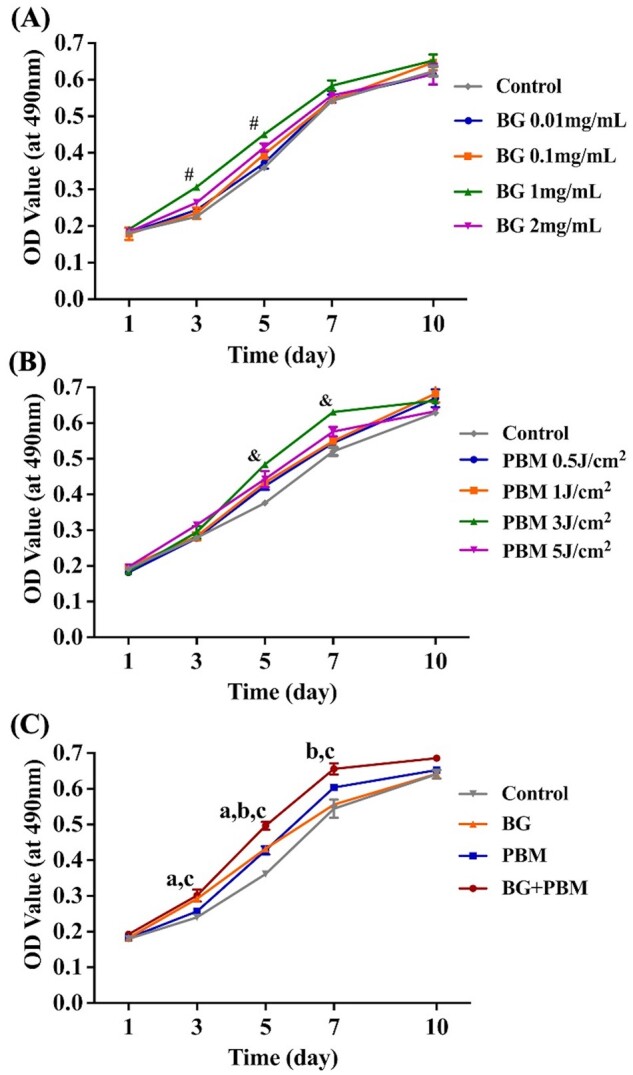
Proliferation assay of hBMMSCs by MTT assay. (**A**) Effects of BG concentrations on hBMMSCs proliferation. (**B**) Effects of PBM fluences on hBMMSCs proliferation. (**C**) Effects of BG combined with PBM on hBMMSCs proliferation. ^#^*P *<* *0.05 comparing the 1 mg/ml group with the control group. ^&^*P *<* *0.05 comparing the 3 J/cm^2^ group with the control group. ^a^*P *<* *0.05 comparing the BG+PBM group with the PBM group; ^b^*P *<* *0.05 comparing the BG + PBM group with the BG group; ^c^*P *<* *0.05 comparing the BG + PBM group with the control group.

The influence of different BG concentrations and PBM fluences on the proliferation of hBMMSCs was detected by MTT assay, respectively. Figure 1A reveals that the OD values of the 1 mg/ml group on Days 3 and 5 were remarkably higher in comparison with those of other groups (*P *<* *0.05), suggesting that 1 mg/ml BG extract concentration promoted hBMMSCs in a more significant way. For the PBM fluence, the group of 3 J/cm^2^ showed the most enhanced hBMMSCs proliferation, with the highest OD value on Day 7 in comparison with other groups (*P *<* *0.05) (Fig. 1B). Based on the above results, the optimum treatment conditions used for the subsequent experiments were determined as 1 mg/ml BG culture medium and 3 J/cm^2^ PBM fluence.

Figure 1C reveals that the OD values of the BG + PBM group were prominently higher than those of the PBM group on Day 3 (*P *=* *0.008) and Day 5 (*P *=* *0.011), the BG group on Day 5 (*P *=* *0.007) and Day 7 (*P *=* *0.001), and the control group on Day 3 (*P *=* *0.017), Day 5 (*P *=* *0.011) and Day 7 (*P *=* *0.015), respectively. The results indicated that BG + PBM displayed additive effects on enhancing the proliferation of hBMMSCs.

### BG combined with PBM promoted hBMMSCs differentiation and mineralization

**Figure 2.**
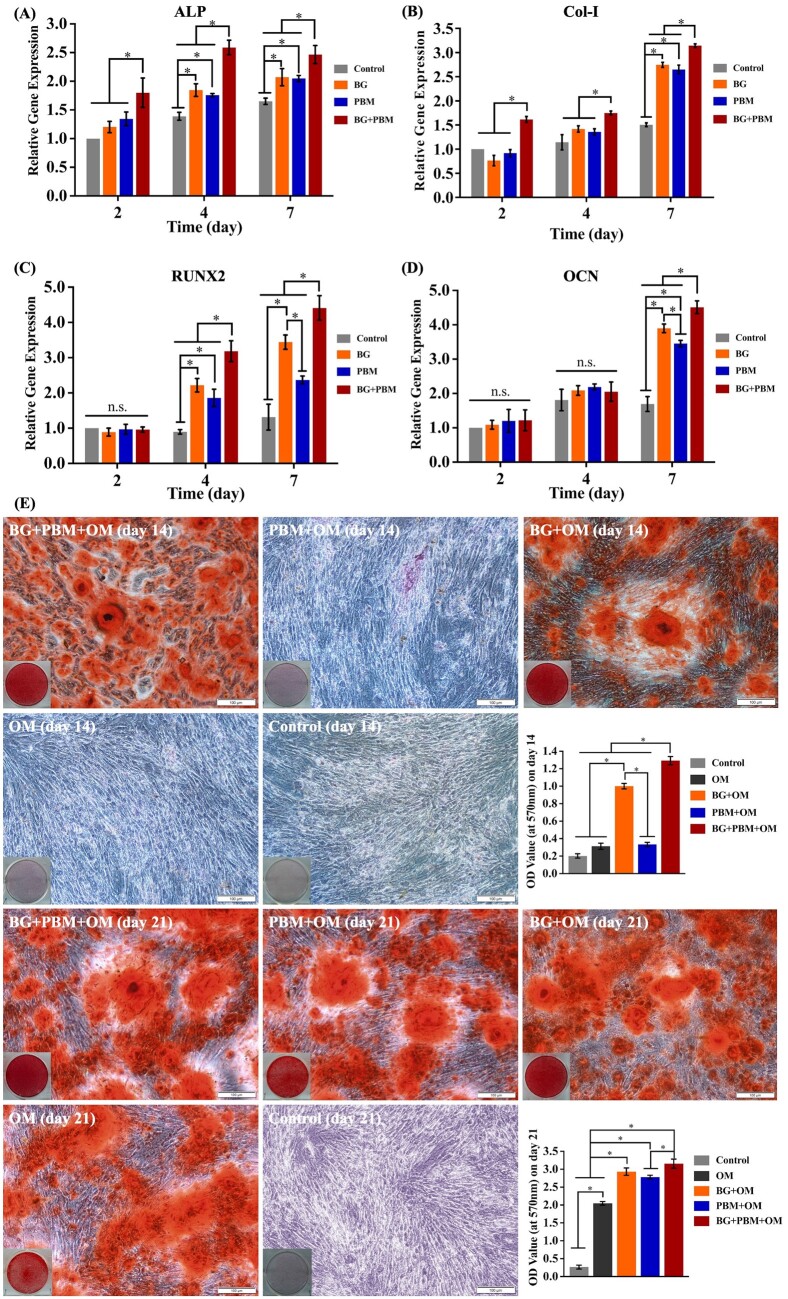
The differentiation and mineralization of hBMMSCs. (**A**–**D**) The mRNA expression of osteogenic-related genes. (**E**) *In vitro* mineralization by alizarin red staining assay and semi-quantitative analysis. *P <* *0.05 between the groups connected by the lines. n.s., no significance among groups.

The genes expression of ALP, Col-I, Runx2 and OCN were remarkably upregulated in BG + PBM group, PBM group and BG group, and mRNA expression in the BG + PBM group was prominently higher than the PBM group and the BG group (*P *<* *0.05) (Fig. 2A–D).

For ALP and Col-I, BG + PBM remarkably promoted the genes expression on Day 2, with significantly higher mRNA levels compared with those of the PBM group and the BG group (*P *<* *0.05). Meanwhile, BG + PBM displayed the most significant enhancement in genes expression (*P *<* *0.05) on Days 4 and 7. For Runx2 and OCN, the BG + PBM treatment revealed the most pronounced up-regulating effect on genes expression. The Runx2 mRNA levels of the BG + PBM group were significantly higher than those of the PBM group on Days 4 (*P *=* *0.025) and 7 (*P *=* *0.011) and were significantly higher than those of the BG group on Days 4 (*P *=* *0.019) and 7 (*P *=* *0.020), respectively. The OCN gene level in the BG + PBM group on Day 7 was significantly higher than those of the PBM group (*P *=* *0.009) and the BG group (*P *=* *0.012), respectively.

The mineralization results (Fig. 2E) displayed that red-stained mineralized nodules formed in the BG + PBM + OM group and the BG + OM group on Day 14, which was earlier than other groups. Compared with the BG + OM group, more mineralized nodules were found in the BG + PBM + OM group. Semi-quantitative analysis revealed that the BG + PBM + OM group displayed the highest OD value (*P *<* *0.05). And the OD value of the BG + OM group was notably higher than those of the PBM+OM group (*P *=* *0.013), OM group (*P *=* *0.007) and control group (*P *=* *0.002).

On Day 21, no mineralized nodules were formed in the control group. A moderate amount of small red-stained mineralized nodules was found in the OM group, while a large number of large red-stained nodules were found in the BG + PBM + OM group, PBM + OM group and BG + OM group. The OD values of the BG + PBM + OM, PBM + OM and BG + OM groups were obviously higher than the OM group and the control group (*P *<* *0.05). The OD value of BG + PBM + OM group was higher than the PBM + OM group (*P *=* *0.028), but there was no statistical significance of the OD values between the BG + PBM + OM group and the BG + OM group (*P *=* *0.082).

### BG combined with PBM promoted new bone formation *in vivo*

HE staining was performed for the observation of tissues formation and biomaterials degradation. The nuclei were blue-purple, while bone tissue and fibrous tissue were red. Masson staining was used to observe the distribution of mature and immature bone matrix in newborn tissues. The bone matrix was dark blue and homogeneous, and the fibrous tissue was light blue. BG particles were dissolved during demineralization and showed transparent irregular blanks after the staining.

**Figure 3.**
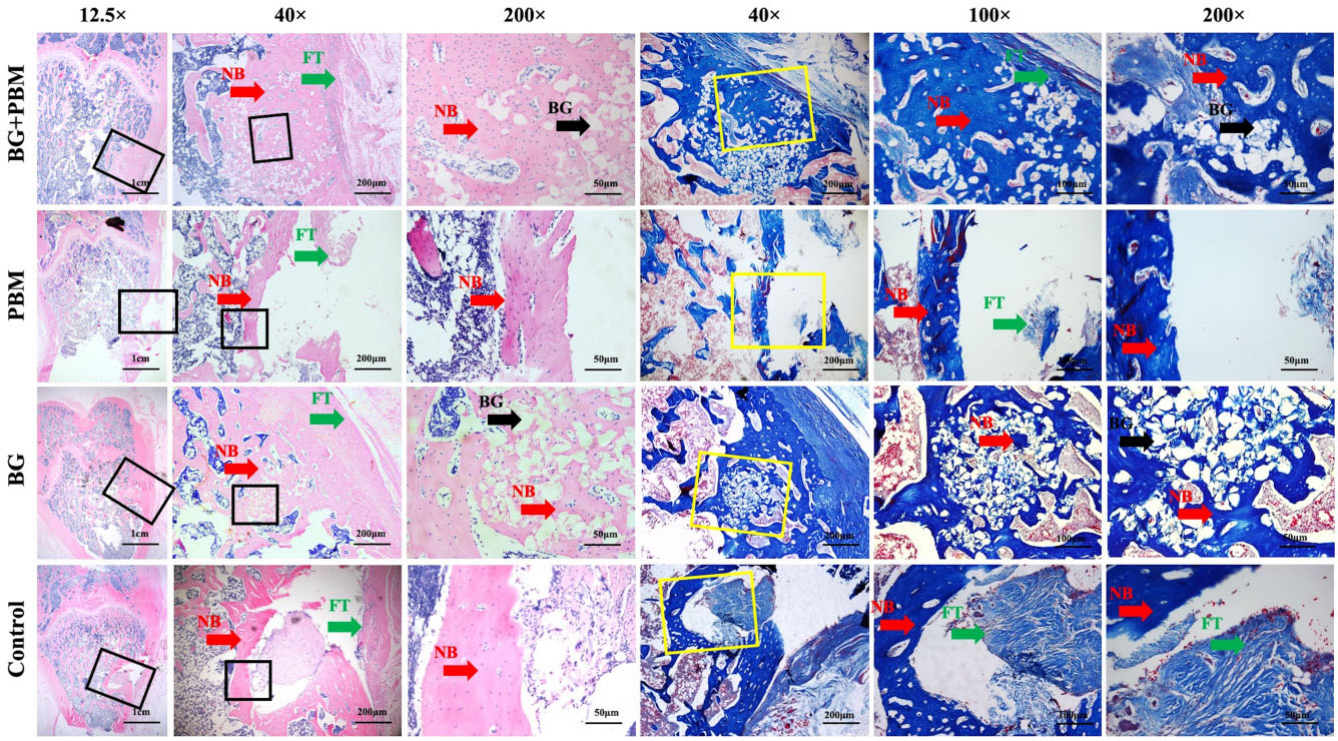
Histological observation by HE staining and Masson’s staining at 6 weeks. The figures on the right are different magnifications of square frames in the left figures. NB, newly bone; FT, fibrous tissues; BG, bioactive glasses.

At 6 weeks post-surgery, tissues in all groups grew well without obvious inflammation (Fig. 3). Both the HE and Masson staining results revealed that, in the PBM + BG group and the BG group, homogeneous red- or blue-stained newly formed bone tissues filled up the whole defects, which were found both on the edge and in the central area (red arrow). Meanwhile, some new bone started to reconstruct and form the lamellar trabecular bone, shown in the figures at 200× magnification. Near the bottom of the defects, some undegraded BG particles formed irregular vacuole structure, which were wrapped by new bone tissues. The edge of the BG particles’ vacuole was irregular and incomplete (black arrow). In the PBM and the control groups, massive new bone tissues mainly distributed on the bottom edge of the defects with no obvious bone remodeling. No new bone was found inside the defects but only some granular tissues.

**Figure 4.**
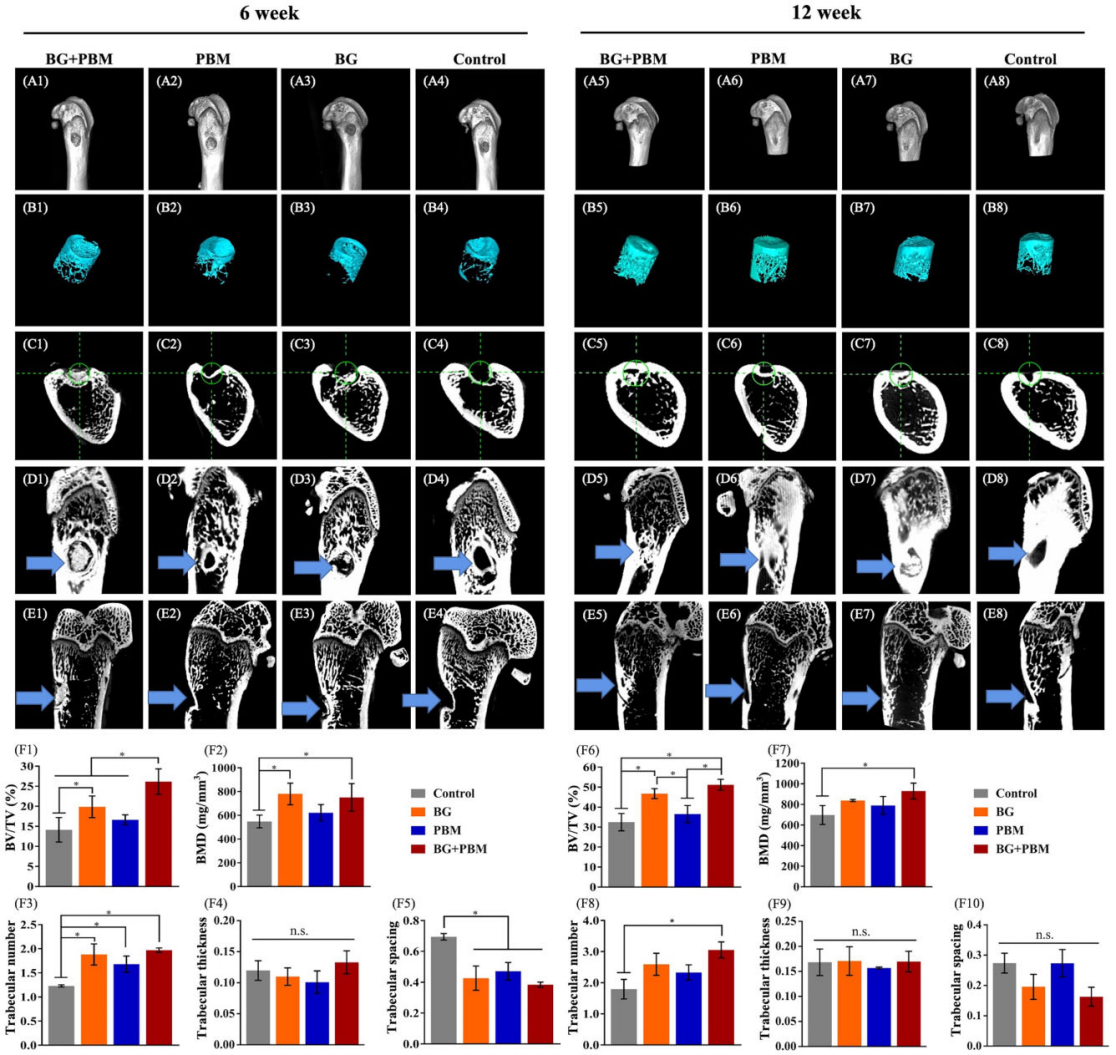
New bone formation *in vivo* by micro-CT at 6 and 12 weeks. (**A**) Scanning of femoral metaphysis. (**B**) Reconstruction of the bone defects. (**C**) Images of femoral metaphysis on cross section. (**D**) Images of femoral metaphysis on coronal section. (**E**) Images of femoral metaphysis on sagittal section. (**F1**–**F5**) Quantitative analysis of micro-CT at 6 weeks. (**F6**–**F10**) Quantitative analysis of Micro-CT at 12 weeks. The circle in (C) is to locate the bone defect on the coronal section. The arrow in (D) and (E) indicates the bone defects on the coronal and sagittal section, respectively. BV/TV, bone volume/tissue volume; BMD, bone mineral density. *P <* *0.05 between two groups. n.s., no significance among groups.

The micro-CT results were shown in Fig. 4, illustrating the new bone formation in each group at 6- and 12-week post-operation. Femoral metaphysis and 3D reconstructed bone defects were shown in Fig. 4A and B. Figure 4C–E displays the images of femoral metaphysis on cross-section, coronal section and sagittal section, respectively, and the blue arrow indicated the location of bone defects and the extent of bone formation. As the results showed that, at 6-week post-surgery, some new bone tissues mixed with partially degraded BG particles were observed inside the bone defects in the BG-treated groups (Fig. 4A1–E1 and A3–E3). BG particles were scatteredly distributed and were irregular in shape. New bone formation was found in all groups. In the BG + PBM group (Fig. 4A1–E1) and BG group (Fig. 4A3–E3), new bone tissues were formed both at the edge and in the interior of the defects, and the reconstruction of the new bone at the edge area could be observed. In the PBM group (Fig. 4A2–E2) and the control group (Fig. 4A4–E4), new bone grew from the edge of bone defects. Quantitative results revealed that the BV/TV value of BG + PBM group was prominently higher in comparison with those of the PBM group (*P *=* *0.009), the BG group (*P *=* *0.026) and the control group (*P *=* *0.003) (Fig. 4F1); the BMD value of samples in the BG + PBM group was significantly higher than that of the control group (*P *=* *0.038) (Fig. 4F2). The BV/TV and BMD of the BG group were also statistically higher than the control group (*P *<* *0.05) (Fig. 4F1 and F2). The BG + PBM group, PBM group and BG group showed notably more trabecular bone formation (*P *<* *0.05) (Fig. 4F3) and significantly less trabecular space (*P *<* *0.05) than the control group (Fig. 4F5), but no statistical significances were observed among the groups of BG+PBM, PBM and BG (Fig. 4F3 and F5).

At 12 weeks post-surgery, large amount of new bone could be observed inside the bone defects of all groups. New bone remodeling was found and a large number of trabecular bones were formed (Fig. 4). The BV/TV values of the BG + PBM group (*P *=* *0.025) and the BG group (*P *=* *0.042) were remarkably higher than that of the PBM group (Fig. 4F6). There were no significant differences of the BV/TV values between the BG + PBM group and the BG group (*P *=* *0.073) (Fig. 4F6). The BG + PBM group was notably higher than the control group for the BMD (*P *=* *0.038) and the number of trabecular bones (*P *=* *0.016), while no statistical differences were observed among the groups of PBM, BG and control (Fig. 4F7 and F8).

## Discussion

Angiogenesis plays a vital role in bone regeneration, as it supplies nutrients to newly formed tissues, eliminates metabolic wastes, delivers growth factors, as well as provides key signals for bone metabolism [25]. The effects of biomaterials on angiogenesis have been confirmed in some studies [26–28]. Nevertheless, the angiogenesis of BG in the early stage was probably not sufficient in the repair of critical-sized defects, which will hamper the new tissue fusion and biomaterials replacement, ultimately leading to the failure of bone repair [12]. The PBM, which is superior on promoting early angiogenesis, may be served as a supplement to BG in bone repair. On the other hand, the osteoinductivity of BG also overcomes the application limitations of the PBM. Our previous study [22] found that the combination of BG and PBM could additively promote early angiogenesis. Therefore, we preliminarily explored their additive effects on promoting bone regeneration in the current study.

The BG used in this study was composed of 22.7% P_2_O_5_–48.2% SiO_2_–29.1% CaO (wt%, named as PSC) and synthesized by the sol–gel technique with phytic acid as phosphorus precursor [23, 24]. This new BG has high phosphorus content, which avoids the sudden increase of OH^−^ releasing in the solution and limits the pH excursion [24, 29]. The pH value of the PSC solution was shown to be stable at ∼7.8 [24]. This moderate and slight pH change reduces the tissue inflammatory reactions that are commonly caused by the strongly alkaline environments [24]. Therefore, PSC can provide stable pH microenvironment for cells’ growth, proliferation and differentiation. In addition, PSC has large specific surface area and can form hydroxyapatite similar to physiological structures, demonstrating high biological activity and tissue-binding capacity [30]. A recent study showed that PSC notably enhanced the proliferation and migration of BMSCs, and also promoted their osteogenic and angiogenic differentiation [31]. Zhu *et al*. [32] also reported that an injectable composite bone cement prepared by PSC significantly promoted bone regeneration in rabbit femoral bone defects. Above all, by providing a stable, cell-friendly pH microenvironment, as well as showing good bioactivity, PSC seems to have a good prospect for improving bone regeneration.

The PBM used in the current study was 808 nm wavelength NIR laser. Lasers of 808 nm wavelength are able to activate mitochondria, increase intracellular calcium, facilitate ATP synthesis and set off a cascade of reactions [33]. Additionally, lasers of 650–950 nm wavelengths can penetrate tissues to a depth of 2–3 mm, which revealed the most enhancing effect on repairing deep defects in comparison with other wavelengths [20, 21].

In the present research, we detected the expression of osteogenic-related genes. As shown in the results, the BG + PBM treatment up-regulated the genes level of ALP and Col-I earlier than the separate application of PBM or BG. Meanwhile, the BG + PBM treatment notably increased the gene levels of ALP, Col-I, Runx2 and OCN, facilitating the differentiation of hBMMSCs into osteoblasts, the maturation of osteoblasts as well as the formation of extracellular matrix. In the alizarin red staining assay, on Day 14, mineralized nodules were observed in the BG + PBM + OM group and the BG + OM group, indicating that BG was able to induce cells maturation and start the mineralization process in advance. On Day 21, the cells matured and the calcium deposition occurred rapidly, so we observed red-stained nodules in all the experimental groups. The BG + PBM + OM group displayed the significantly higher OD values at both timepoints, suggesting that, in comparison with the separate application of PBM or BG, the BG + PBM treatment initiated the hBMMSCs’ extracellular matrix mineralization earlier and notably promoted the degree of mineralization. Above results indicated that the combined application of BG and PBM generated an additive effect, which promoted the expression of osteogenic-related genes earlier, significantly enhanced the osteogenic differentiation and mineralization, and rapidly initiated the osteogenic process. The pro-osteogenic effect of BG combined with PBM was better than applying BG or PBM singly.

We further observed the effect of BG combined with PBM on bone defect repair *in vivo*. The results of histological staining showed that BG induced new bone tissue to grow inside the materials, and the new bone started to reconstruct at 6 weeks after surgery. However, in the groups without biomaterials, new bone mainly formed at the edge of the defects. Micro-CT results showed that BG + PBM treatment significantly increased the volume of new bone, the density of bone mineralization and the number of trabecular bones at 6 weeks post-operation. At 12 weeks, the BMD and the number of trabecular bones in the BG + PBM group were prominently higher than that of the control group, while no significant differences were observed among the groups of PBM, BG and control. Above results indicated that BG promoted the formation of new bone tissue. Comparing with BG or PBM, BG combined with PBM accelerated bone formation and maturation, improved the speed and quality of bone regeneration, as well as facilitated the repair of bone defects. The effect was better than applying BG or PBM singly.

The mechanism for these additive effects exerted by BG combined with PBM may be 2-fold. Firstly, both BG and PBM can activate the cell signal pathways of osteogenesis and angiogenesis, and promote early angiogenesis and bone regeneration, respectively [16, 19, 28, 34–36]. For one thing, BG releases appropriate concentration of Si, Ca and P ions to promote cell proliferation and differentiation, and accelerate bone regeneration and remodeling [37]. Si can enhance ALP activity, activate Wnt-β-catenin and some other signal pathways, upregulate the expression of OCN, OPN and Runx2 genes, and promote the osteogenic differentiation of hBMMSCs [38, 39]. The increase of extracellular Ca can regulate the proliferation and differentiation of osteoblasts by enhancing Ca releasing from intracellular calcium stores and activating a series of signal pathways such as MAPK pathway [40]. In addition, extracellular Ca can upregulate intracellular Ca concentration through l-type calcium channel, activate CaM-CaMK2α and ERK1/2 signal pathways, increase the expression of BMP2, BSP and OPN and promote osteogenesis [40–42]. P can activate ERK1/2 and PKC signal pathways, improve ALP activity and increase OPN content [43]. For another, PBM has been widely believed to stimulate cytochrome c oxidase, change mitochondrial membrane potential, increase intracellular Ca concentration, and promote ATP synthesis [33, 44]. Additionally, NIR lights can activate light-sensitive gated ion channels and increase the levels of intracellular Ca, which then interacts with reactive oxygen species and cyclic AMP [45]. These changes have been delivered to the nucleus, further upregulating genes expression by activating AP-1, NF-kB transcription factors and promoting cell proliferation and differentiation [33]. Whether there is interaction between BG and PBM still needs to be confirmed. From the subcellular level, it is speculated that the additive effect of BG and PBM may be related to the increase of cytoplasmic free Ca concentration. Then, cytoplasmic free Ca may serve as a second messenger to activate a series of downstream signal pathways. The signal pathways may interact and promote each other, and then upregulate related genes expression, further promoting cell proliferation, differentiation and other physiological activities. In future studies, we still need to explore and examine the additive mechanism of BG and PBM at the subcellular and molecular levels.

Secondly, the enhanced early angiogenesis may promote bone regeneration and accelerate bone defect healing. In our recently published paper, we used BG and PBM in combination to observe their effects on angiogenesis [22]. We found that the superiority of PBM on angiogenesis could overcome the inadequacy of BG in early angiogenesis, and the combination of both would generate additive effects on enhancing human umbilical vein endothelial cells’ proliferation, angiogenic-related growth factors’ gene expression and tubules formation *in vitro*, as well as promoting early angiogenesis *in vivo* [22]. Therefore, the sufficient newly blood provided by PBM and BG on the early stage will supply rich nutrients to cells and tissues, which will further enhance the cell activities and promote the bone repair.

Besides enhancing osteogenesis, the additive effect of BG combined with PBM also has advantages in biological safety by reducing the use of chemicals or growth factors. Compared to adding proangiogenic microelements into BG particles, the combined application of PBM avoids biosafety issues caused by the microelements’ concentration and release rate [46]. Moreover, the results of our previous study showed that PBM could reduce the inflammatory reaction caused by materials implantation [22], which can avoid excessive harmful inflammation damaging cells, and facilitate an environment conducive to tissue regeneration [47]. Therefore, it’s feasible to combine BG and PBM in bone tissue engineering. The additive effects of BG and PBM on osteogenesis together with the beneficial effects of PBM on improving biocompatibility enable their promising clinical applications in the bone defect repair.

Recently, some studies combined biomaterials with PBM and have achieved positive results in tissue regeneration [48–50]. However, some other studies showed that the superiority of PBM cannot be exploited when combined with biomaterials, which may be related to the excessive stimulation of tissue caused by the inappropriate dosage of biomaterials and PBM [51]. Therefore, our research also highlighted the significance of doses in the application of BG and PBM. For the application of PBM therapy, we selected the optimum fluence of PBM *in vitro* at first. And we conducted irradiation intermittently to avoid the overload of intracellular calcium that may exhaust the cells’ energy reserve and even cause the cells’ death. The same was also true of our experimental results that cell growth could only be enhanced with appropriate dosages. As for the PBM dose of the *in vivo* study, we referred to the previous literature in which 140 J/cm^2^ had been reported to generate beneficial effects on bone repair [52], as well as the results in our pro-angiogenic study where 120 J/cm^2^ had been used *in vivo* [22]. We ultimately selected 120 J/cm^2^ as the fluence on the basis of safety consideration. Additionally, considering the energy attenuation caused by the blocking of skin and muscles [21], as well as the fact that the number of mitochondria within bones and bone marrow is relatively lower [52], we need more energy to exert PBM efficacy. So, higher doses of energy should be applied *in vivo* for stimulating mitochondrial activity and promoting tissue regeneration. The fluence of *in vivo* study was much higher than that of the *in vitro*.

For the application of BG, we also concerned about the BG concentration. We performed the pre-experiment to obtain the optimum BG concentration for the following *in vitro* experiments. Another concern was the environmental pH changes due to the alkaline ions ‘burst release’ when biomaterials rapidly exchange ions with the surrounding medium [53]. Therefore, we performed the 24-h pre-incubation of BG *in vitro* during the preparation of the BG conditioned medium.

Although we have achieved some positive effects in the present study, there are still several limitations. Firstly, the optimal parameters of PBM therapy used *in vivo* still need more studies, especially applied with biomaterials in combination. The dosages may vary with different laser types and wavelengths. Secondly, the transmitted power of light varies with different tissue thickness and anatomical structures, and the light energy attenuates with the increase of tissues depth [54–56]. So, the loss of light energy in tissues should be taken into account when determining the PBM doses *in vivo*, and appropriate dosages should be selected to ensure that the full depth of bone defects absorb sufficient energy. Thirdly, the flat-top hand-piece should be applied instead of the standard Gaussian probe in future studies to ensure the consistency of PBM efficacy [57].

## Conclusion

The present study showed that the combination of BG and PBM exerted additive effects on enhancing hBMMSCs’ proliferation, osteogenic-related genes expression and mineralization *in vitro*, as well as accelerating *in vivo* bone regeneration and maturation by improving the speed and quality of new bone formation. The advantages of PSC on its good osteoinductivity and the stable, cell-friendly pH change of microenvironment, cooperated with the beneficial effects of PBM on improving biocompatibility and angiogenesis, enabled their good application prospect in the bone repair. Whether the dosage of PBM and BG was appropriate played an important role in the cells’ growth and tissue regeneration. Therefore, more efforts should be made in the fields of determining the optimum dosages of BG and PBM to amplify their additive effects, and exploring appropriate *in vivo* strategies to provide a foundation for their clinical application.
